# XEN® stent complications: a case series

**DOI:** 10.1186/s12886-019-1267-y

**Published:** 2019-12-12

**Authors:** Chandni Gupta, Divya Mathews

**Affiliations:** Stanley Eye Unit, Abergele Hospital, Llanfair Road Abergel Conwy, Abergele, Wales LL22 8DP

**Keywords:** XEN, Glaucoma, MIGS

## Abstract

**Background:**

XEN® gel stent (Allergan, Dublin/Republic of Ireland) is a relatively new microinvasive glaucoma device providing an ab-interno approach to the subconjunctival space for aqueous drainage and reduction of intraocular pressure. It is thought to be less invasive, reduce surgical time and post-operative infection rates compared with traditional glaucoma procedures. Little information however, has been published regarding complications and subsequent management.

**Case presentation:**

The authors highlight five complicated cases of XEN® stent insertion, how they were managed and key learning points. Cases include: entire stent found at the bottom of the anterior chamber several months after uncomplicated insertion, stent broke into multiple pieces during manipulation within subconjunctiva, XEN45 stent migrated into the anterior chamber 7 months post-operatively and a case of limbal-based conjunctival dissection during open revision which lead to additional scarring around the stent and subsequent raised intraocular pressure.

**Conclusions:**

We present some new and interesting complications of XEN implant as well as potential management options. This can assist clinical decision-making and enable better pre-operative discussions with patients regarding risks of surgery.

## Background

The XEN45® (XEN) stent is a 6 mm long implant made from porcine gelatine, manufactured by Allergan (Dublin/Republic of Ireland). It is classed under the umbrella term Minimally Invasive Glaucoma Surgery (MIGS) as it is thought to be less invasive, reduce surgical time and post-operative infection rates compared with traditional glaucoma procedures [1–3]. The stent is designed to drain aqueous fluid from the anterior chamber to the subconjunctival space, thereby reducing intraocular pressure (IOP) [4]. It is injected via an ab-interno approach; and although the conjunctival surface does not require dissection with the added advantage of reduced likelihood of infection, the procedure benefits from the use of Mitomycin C (MMC) to prevent scarring.

Clinical experience with XEN stents is still within its infancy and therefore also is the knowledge of its subsequent safety and efficacy profile [5, 6]. In our unit we have carried out over 50 XEN stent insertions. In this article we highlight some of the interesting complications encountered, and subsequent post-operative measures taken.

### XEN implantation procedure

In our unit, XEN stent insertion is carried out under subtenons anaesthetic block with 3-5mls of 2% Lidocaine and Hyaluronidase. Following adequate anaesthesia 0.01% in 0.1 ml of MMC is injected posteriorly within the subconjunctival space and massaged forwards, towards the desired area for implantation (usually superonasal quadrant). The conjunctiva is then marked 3 mm from the limbus superonasally; where the tip of the stent is expected to exit from the scleral tunnel created. An inferotemporal clear corneal incision is made and Healon GV® (Abbott Medical Optics Inc., California/United States) is injected to fill the anterior chamber (AC). Additionally a paracentesis wound is created three clock hours away from the main incision. This site is used to manipulate the eye into the correct position via a second instrument. A pre-loaded 27 gauge needle is used to insert the XEN implant ab-interno, via the inferotemporal clear corneal incision, and advanced to the superonasal part of the AC under gonioscopic view. The stent is delivered anteriorly to the trabecular meshwork and into the subconjunctival space. The viscoelastic is then aspirated from the AC and the patient is given stat 0.1mls intracemeral Cefuroxime (3 mg in 0.3mls) and 1 ml subconjunctival Dexamethasone (3.3 mg in 1 ml). All patients in this unit routinely receive Gutte Prednisolone acetate 1% four times/day and Gutte Chloramphenicol 0.5% four times/day post-operatively for 4 weeks.

Ideally the 6 mm stent should have 3 mm within the subconjunctival space, 2 mm within the scleral tunnel and 1 mm in the AC. If combined with cataract surgery, XEN stent insertion is performed after completion of cataract surgery and constriction of the pupil with Miochol®-E (Bausch & Lomb Inc., New York/United States) [2].

### Case presentation

#### Case 1

A 60 year old gentleman with advanced primary open-angle glaucoma (POAG) underwent uncomplicated right eye XEN stent insertion with MMC. His pre-operative vision was 0.24 LogMAR, and IOP 13 mmHg on three IOP-lowering eye drops. Two weeks following surgery the IOP had risen to 38 mmHg and tenons tissue was noted to be blocking the subconjuctival stent tip. Needling with 5FU was performed in clinic. Although the IOP reduced to 32 mmHg this was inadequate, hence the patient underwent further open revision with 5FU in theatre through a 3–4 mm incision a week later. A limbal-based conjunctival approach was used to dissect the stent from the tenons; as it was deeply embedded, and was successful.

**Fig. 1:**
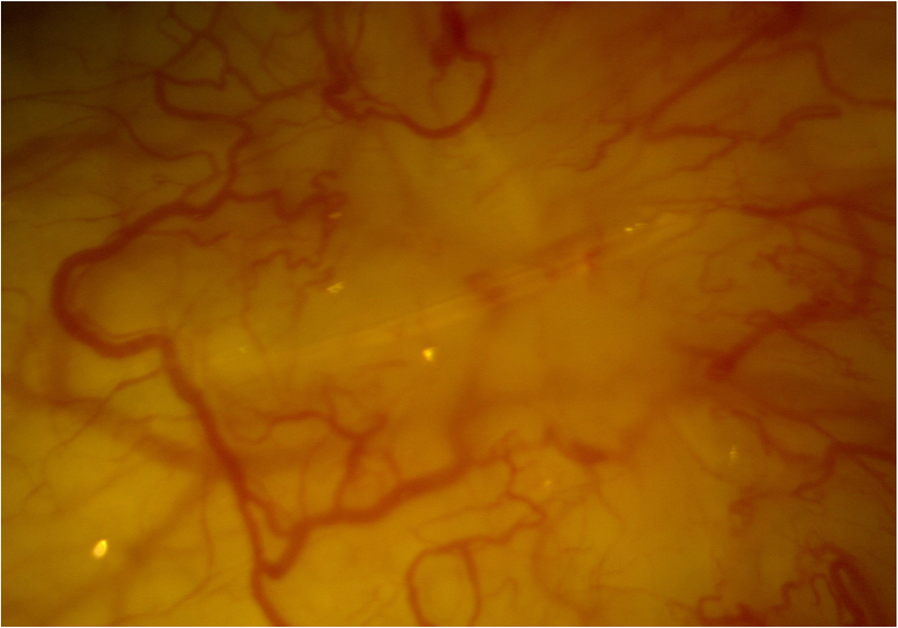
Distal end of XEN stent exposed in right eye

One day post-operatively the IOP had reduced to 10 mmHg, with a well formed anterior chamber. The bleb however, was noted to be shallow. The patient continued with his routine post-operative drops and was seen again a week later when it was noticed that the subconjunctival end of the stent was exposed through a gape in the conjunctiva from the revision site (Fig. 1). The patient was again taken to theatre where the conjunctiva was closed with 8/0 vicryl. Despite this, the overlying conjunctiva continued to breakdown and re-expose the stent. As a final measure, the tip of the exposed stent was purposefully broken in clinic so that the remainder could slide under the conjunctiva and enable healing. Since this manoeuvre, the conjunctiva has healed well and the stent is no longer exposed. The patient continues to be monitored however, is now also on two pressure-reducing drops to the affected eye and maintaining an IOP of 17 mmHg.

Learning Point: Consider a fornix-based conjunctival dissection, rather than a limbal, as this reduces the risk of scarring directly around the stent. In addition, do not consider repeating the use of anti-metabolites so soon after initial stent insertion as this can impair necessary tissue healing after the procedure.

#### Case 2

A 64 year old gentleman with poor baseline vision (RE: count fingers, LE: 1.34 LogMAR); due to advanced glaucoma as well as longstanding chorioretinal scarring, had combined phacoemulsification and XEN stent with MMC to the left eye. His pre-operative intraocular pressure was 13 mmHg on four IOP-lowering medications.

During the surgery the initial stent could not be seen within the subconjunctival space or the AC. It was thought that perhaps the entirety of the stent had entered the supraciliary space or had become intrascleral therefore a second stent was inserted. One day post-operatively the vision improved (1.06 LogMAR) and the IOP remained well controlled at 13 mmHg, without any glaucoma medications. When seen again 5 weeks post-operatively his vision had reverted back to that of pre-surgery and his IOP had risen to 23 mmHg. Querying whether he was a steroid responder, Gutte Prednisolone acetate 1% was stopped. Three weeks later the pressure had lowered back to 12 mmHg, and the stent was noted to be insitu.

**Fig. 2:**
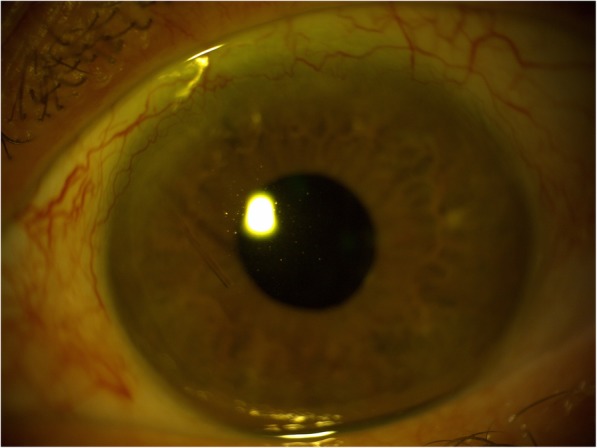
XEN stent migrating into the anterior chamber of the right eye

**Fig. 3:**
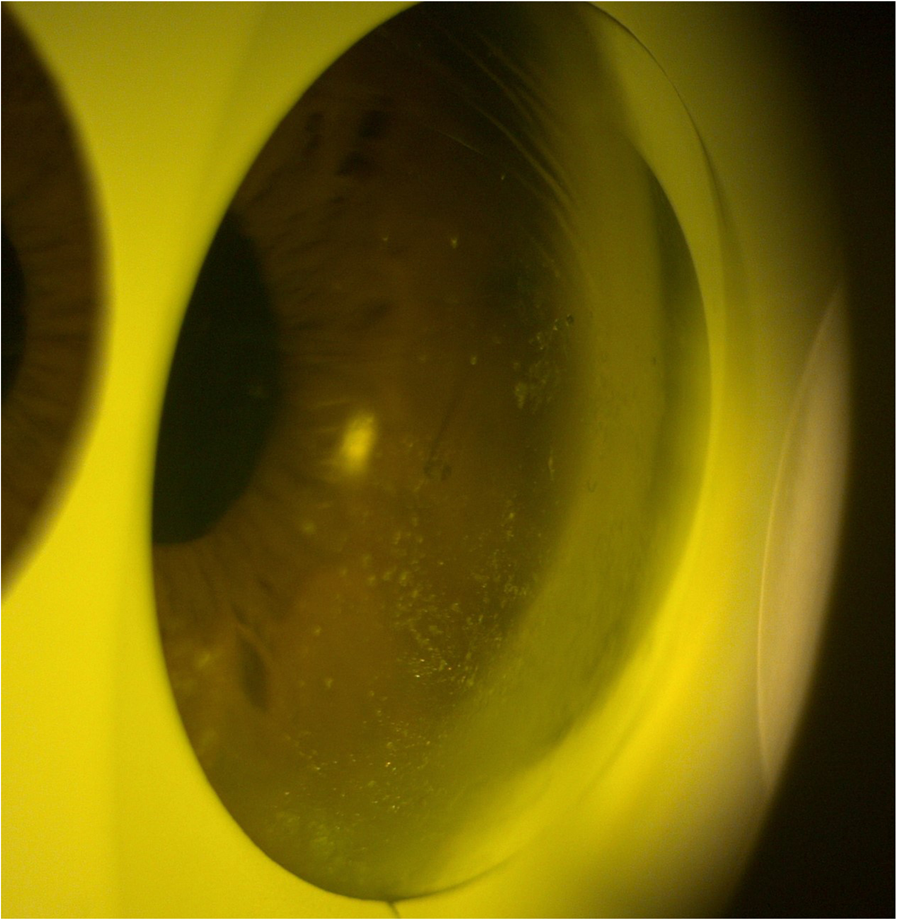
Gonioscopic examination showing migrated XEN stent

Roughly 7 months later when seen at routine follow-up, slit lamp biomicroscopy revealed the initial ‘lost’ stent had migrated back into the anterior chamber (Figs. 2 and 3). At this stage the vision was stable and there were no concerns with regards to IOP. The patient was listed for removal of stent due to risk of corneal decompensation secondary to the stent coming into contact with the corneal endothelium. The second stent has remained insitu with no adverse effects.

Learning Point: XEN stents can migrate from their initial insertion point therefore it is important to perform gonioscopic examination on a regular basis at follow-up appointments. Additionally, if the displaced stent is likely to come into contact with the cornea, consider removal.

#### Case 3

A 77 year old male with primary open angle glaucoma had left eye combined cataract and XEN stent surgery with MMC. Listing best corrected visual acuity was 0.32LogMAR, and the IOP 12 mmHg on two pressure-reducing eye drops. Intra-operatively the stent had to be re-sited due to suboptimal positioning during initial placement. This was followed by moderate haemorrhage into the anterior chamber and subconjunctival space, which later lead to post-operative scarring around the implant. Multiple needling attempts with 5FU were carried out in clinic but failed. This was due to recurrent tenons tissue encapsulating the stent, leading to blockage.

Almost 6 months after the initial procedure the IOP had risen to 18 mmHg. It was noted that the bleb was flat and the stent not visible, so the patient was listed for bleb revision. During surgery, whilst dissecting the tenons tissue from the stent, the implant broke and therefore a new XEN stent was inserted.

Learning Point: In cases where bleeding into the AC and/or subconjunctival space has occurred, one must be mindful of the potential of early scarring. It may be more appropriate in these circumstances to perform needling with 5FU in theatre as these cases will have more tenons tissue encapsulating the stent. When severe encapsulation is encountered, one must bear in mind the possibility of needing to insert a new stent if the original breaks during manipulation. It might be advisable to obtain consent and council patients for this complication prior to surgery.

#### Case 4

A 79 year old gentleman had routine left eye combined cataract and XEN stent insertion with MMC. His pre-operative visual acuity was 0.24LogMAR and IOP 20 mmHg on 3 pressure-reducing medications. His post-operative intraocular pressures were maintained between 13 and 14 mmHg until roughly 11 months when topical medications had to be reinstated. Despite this however, his IOP gradually continued to rise. It was queried whether his implant was working therefore an attempt was made to straighten the stent and free some tenons tissue from the tip during a clinic visit. This resulted in a 3 mmHg drop in intraocular pressure. His IOP continued to be stable and implant well positioned at subsequent visit 6 weeks later.

**Fig. 4:**
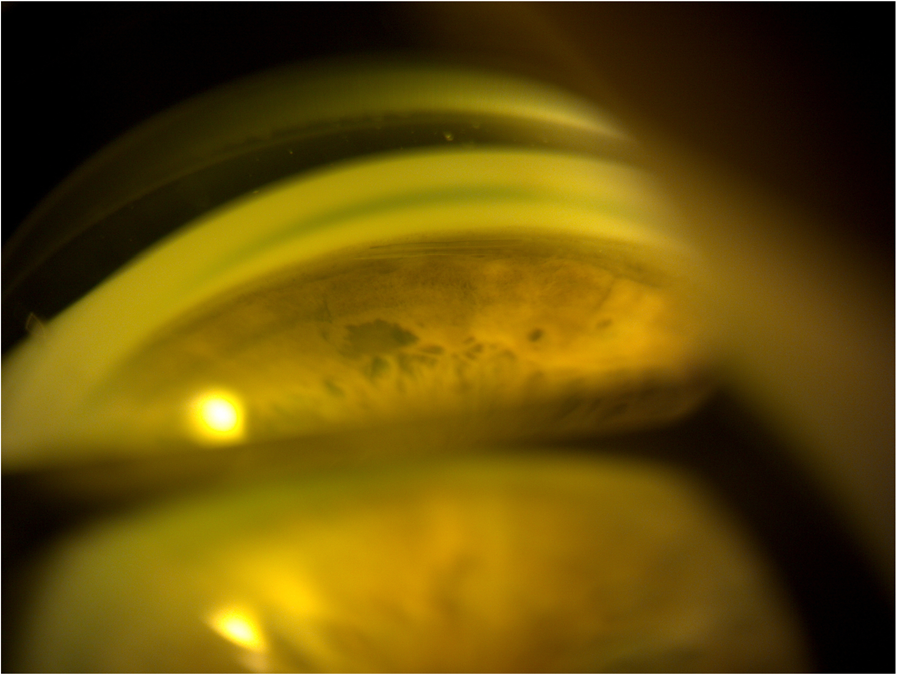
Gonioscopic view showing the entirety of the XEN stent lying at the bottom of the anterior chamber

When seen during a follow-up visit 3 months later the entire XEN stent was found lying at the bottom of the anterior chamber (Fig. 4). A good bleb was seen superonasally, indicating that the channel from the AC to the subconjunctival space had been maintained. As the implant is inert and not likely to come into contact with the cornea it was deemed safe to leave the implant in the angle.

The patient later explained that due to his hayfever, he had been rubbing his eyes quite vigorously for the last few weeks.

Learning Point: Patients must be counselled against ‘strenuous rubbing’ of eyes as this can displace the implant; even several months following insertion.

If the entire XEN stent dislocates there is a possibility that the channel between the AC and subconjunctiva will be maintained and therefore, continued control of IOP may be achieved as in this case.

#### Case 5

An 82 year old gentleman had combined right eye cataract surgery with XEN stent insertion and MMC for advanced primary open angle glaucoma. His pre-operative visual acuity was − 0.08 LogMAR and IOP 20 mmHg on 4 pressure-reducing eye drops. He had already had left eye combined cataract surgery with viscocanalostomy in the left eye, and was not using any topical medication.

**Fig. 5:**
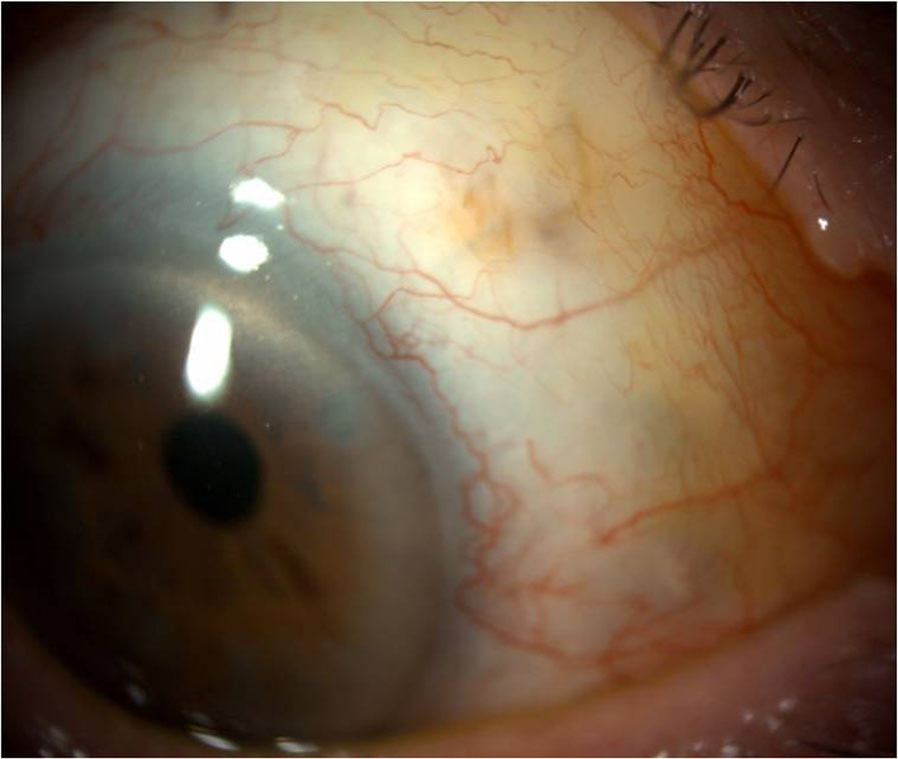
XEN stent fragments within subconjunctival space; sitting adjacent to newly inserted stent

At the time of stent insertion it was queried whether the subconjunctival end of the stent was too short however, due to a good bleb being achieved on the table; indicating good drainage, it was decided not to reposition the stent. One day post-operatively it was noted that the tip was tenting beneath the bleb therefore the stent was pulled from the subconjunctival end in clinic. Several attempts were made to position the implant correctly and achieve a 3 mm length subconjunctivally. However during manipulation, it was noted that the implant had fragmented into multiple pieces within the subconjunctiva space. The patient was listed for further XEN stent and MMC, which was inserted adjacent to the initial implant, with no complications (Fig. 5). The fragmented pieces were not removed.

Almost 2 years since the second stent surgery the patient remains on no glaucoma medications and has maintained his vision at − 0.06 LogMAR and IOP at 12 mmHg.

Learning Point: If during the procedure there is any doubt regarding positioning of the XEN, it is best to re-position at the same sitting. The stent is very fragile therefore manipulation can result in breakage. This should be bourne in mind during the procedure.

### Discussion and conclusion

Whilst intraoperative complications and the subtleties regarding stent insertion are becoming better understood, information on the long term performance and behaviour of these stents is still lacking. Although several papers discuss the complication statistics associated with XEN stent insertion, few discuss the subsequent real-world surgical practicalities regarding their management [2, 5, 7]. This small case series aims to highlight some interesting complications, as well as possible management options. We discuss some of the learning points below.

MMC is used routinely as part of the XEN stent insertion procedure however should be used cautiously; even where there is initial scarring. It must be noted that there are currently no randomised-control trials assessing the long term efficacy or safety profile when MMC is used as opposed to when it is not during XEN stent insertion, although it has been stated for other ophthalmic procedures [6]. There is also no study looking into whether this procedure results in the presence of MMC in the anterior chamber and the effect of this. The above may be something to consider given that the amount and concentration of MMC used during XEN stent implantation differs slightly among ophthalmic units [4, 5, 7].

Haemorrhage into the AC or subconjunctival space can also lead to unwanted scarring and tenons encapsulation of the stent. Our authors would recommend performing needling only if the stent can be visualised beneath the conjunctiva. If this is not possible then we would consider other options such as bleb revision. The aim of needling is to free the stent from the tenons tissue so that it is mobile, with a surrounding low and diffuse bleb [8].

Ensure patients with allergic eye disease are treated appropriately prior to surgery to ensure minimal rubbing of eyes and thus prevent stent migration and subsequent sequelae. Although we are not sure whether rubbing of the eye was the primary trigger factor for this complication it is likely to have contributed to stent migration. A similar case was reported by Denervis et al. who suggested a change in XEN stent design to prevent dislocation; such as progressively increasing the lumen width [9].

Should there be any concern with regards to the positioning of the stent intra-operatively it is suggested to re-position, or insert a new stent, at the same sitting. XEN stents are delicate and manipulations can lead to breakage. To prevent re-listing the patient back and therefore utilising valuable theatre time we would suggest managing the situation immediately. A recent case report by Lapira et al. discussed extrusion and breakage of XEN stent, culminating in endophthalmitis [10].

In our unit we have carried out over 50 XEN stent implantations and have progressively changed our insertion technique and post-operative management based upon our experiences. The above learning points are to be used to complement formal XEN stent training, and help in counselling patients fully with regards to operative complications.

## Data Availability

Not applicable.

## Abbreviations

5FU: Fluorouracil
AC: Anterior Chamber
BCVA: Best Corrected Visual Acuity
IOP: Intraocular Pressure
MIGS: Minimally Invasive Glaucoma Surgery
MMC: Mitomycin C
POAG: Primary Open-Angle Glaucoma
XEN: XEN45® (Allergan, Dublin/Republic of Ireland)
